# Ambulances in the City

**Published:** 1905-04-29

**Authors:** 


### AMBULANCES IN THE CITY.

An interesting report from the Police Committee of the City of London to the Court of Common Council, on the subject of Horse Ambulances, which was presented on the 30th ultimo, has now been published, and contains a very strong recommendation that a supply of these conveyances should be forthwith established and brought into proper working order. It sets forth that the annual average of street accidents removed by the City police to hospitals during the last five years has been 1,952, or between six and seven daily for the working days of the year, and it suggests that the necessary provision for dealing with these accidents in a proper manner, with regard alike to the treatment of the injured and to the prompt removal of obstructions from the thoroughfares, would be met by two ambulance stations, serving respectively the western and the eastern portions of the city. Cases from the western district would be conveyed to St. Bartholomew's, and those from the eastern district to Guy's or to the London hospitals. Details are given as to the men, horses, and carriages that would be required; and it is shown that the cost of the proposed establishment would be £1,184 per annum. The committee has had under consideration the question of the expediency of employing mechanically-propelled ambulances, and also the cost of such a service; but, having due regard to medical opinion concerning the vibration of the conveyances, and to the facts that while a motor ambulance, so far as they can ascertain, has not yet been successfully worked anywhere (in New York the motor has been discarded and horse ambulances reverted to) the committee think that the use of an electric motor in the city would render the success of the undertaking very doubtful, and they therefore recommend the use of horse traction as proposed by the Commissioner. The report concludes by expressing the opinion that "it is most desirable that the horse-ambulance system proposed by the Commissioner be adopted in its entirety," and by recommending that it "should be referred back to us to give the necessary directions to carry out the same." The City, under its local Acts referring to "obstructions," has full power to deal with the matter as it may think fit; and it may therefore be hoped that an ambulance service will soon be provided within its boundaries, and that a good example will thus be afforded to the several outlying municipalities of which London as a total is composed.

